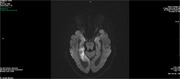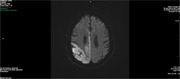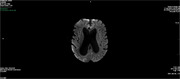# Recurrent Strokes and Neurologic Decline: When to Suspect MELAS Syndrome

**DOI:** 10.1002/alz70855_106536

**Published:** 2025-12-24

**Authors:** Sarika Mutyala, Laith N Maali, Sai Kumar Pasya

**Affiliations:** ^1^ Gandhi Medical College, HYDERABAD, Telangana, India; ^2^ University of Kansas, Lawrence, KS, USA; ^3^ University of Kansas, Manhattan, KS, USA

## Abstract

**Background:**

Mitochondrial encephalomyopathy, lactic acidosis, and stroke‐like episodes (MELAS) syndrome is a rare mitochondrial genetic disorder primarily caused by the m.3243A>G mutation in the *MT‐TL1* gene. It is marked by defects in mitochondrial translation, nitric oxide deficiency, and reduced energy production, resulting in multiorgan dysfunction. Standard clinical features include recurrent stroke‐like episodes, hearing loss, seizures, and various neurological impairments. The primary goals of treatment are to manage disease complications and enhance the patient's quality of life.

**Method:**

A 56‐year‐old man with a history of bilateral hearing loss since his 20s and mild hypertension presented with left visual field defects. MRI revealed a right occipital infarct, prompting initiation of dual antiplatelet therapy, later switched to anticoagulation due to a heterozygous prothrombin gene mutation. Over the subsequent two months, he experienced a recurrent, stepwise neurological decline, including worsening left‐sided weakness, subjective cognitive impairment, and functional deterioration. Repeat brain MRIs demonstrated progressive cortical hyperintensities on DWI and FLAIR sequences in the right posterior brain regions. Further evaluation revealed persistently elevated serum and CSF lactate, elevated pyruvate, and increased serum alanine. Based on the clinical progression and biochemical abnormalities, MELAS was suspected, with genetic testing pending confirmation. Mitochondrial‐targeted supplements, including riboflavin, CoQ10, and B vitamins, were initiated, and atorvastatin was discontinued due to potential mitochondrial toxicity. Hematology consultation recommended stopping anticoagulation therapy.

**Result:**

The patient exhibited a progressive neurological decline with recurrent stroke‐like episodes over two months despite appropriate anticoagulation and antiplatelet therapy and cognitive decline. MRI showed a right occipital infarct, and lab results revealed increased serum and CSF lactate and pyruvate levels. Given the biochemical abnormalities and radiological findings, MELAS syndrome was suspected, and appropriate management was initiated.

**Conclusion:**

This case highlights the importance of considering mitochondrial disorders in patients with recurrent strokes, mainly when imaging and clinical progression are atypical for a vascular etiology. Timely recognition and metabolic testing are essential for effective management. This report emphasizes the significance of early intervention in improving outcomes for patients with MELAS.